# A Comparison of Two Methods for MRI Classification of At-Risk Tissue and Core Infarction

**DOI:** 10.3389/fneur.2014.00155

**Published:** 2014-09-03

**Authors:** Richard Leigh, Victor C. Urrutia, Rafael H. Llinas, Rebecca F. Gottesman, John W. Krakauer, Argye E. Hillis

**Affiliations:** ^1^Johns Hopkins University School of Medicine, Baltimore, MD, USA

**Keywords:** MR RESCUE, DEFUSE 2, MRI, penumbra, DWI, PWI, at-risk tissue, core infarction

## Abstract

**Objective**: To compare how at-risk tissue and core infarction were defined in two major trials that tested the use of MRI in selecting acute stroke patients for endovascular recanalization therapy.

**Methods**: MRIs from 12 patients evaluated for possible endovascular therapy were processed using the methods published from two major trials, MR RESCUE and DEFUSE 2. Specifically, volumes of at-risk tissue and core infarction were generated from each patient’s MRI. MRIs were then classified as whether or not they met criteria for salvageable tissue: “penumbral pattern” for MR RESCUE and/or “target profile” for DEFUSE 2 as defined by each trial.

**Results**: Volumes of at-risk tissue measured by the two definitions were correlated (*p* = 0.017) while the volumes of core infarct were not (*p* = 0.059). The volume of at-risk tissue was consistently larger when defined by the penumbral pattern than the target profile while the volume of core infarct was consistently larger when defined by the target profile than the penumbral pattern. When these volumes were used to classify the MRI scans, 9 out of 12 patients (75%) were classified as having a penumbral pattern, while only 4 out of 12 patients (33%) were classified as having a target profile. Of the 9 patients classified as penumbral pattern, 5 (55%) were classified differently by the target profile.

**Interpretation**: Our analysis found that the MR RESCUE trial defined salvageable tissue in a way that made it more likely for patients be labeled as favorable for treatment. For the cohort of patients examined in this study, had they been enrolled in both trials, most of the patients identified as having salvageable tissue by the MR RESCUE trial would not have been considered to have salvageable tissue in the DEFUSE 2 trial. Caution should be taken in concluding that MRI selection for endovascular therapy is not effective as imaging selection criteria were substantially different between the two trials.

## Background

In the stroke literature, the term “penumbra” was originally introduced to describe brain tissue that is electrically dysfunctional due to inadequate blood flow (1). The term was subsequently adopted by the MRI literature to reflect “at-risk” tissue estimated by diffusion–perfusion mismatch (DPMM). It is theorized that tissue that does not have restricted water movement on diffusion weighted imaging (DWI) but does have disrupted blood delivery on perfusion weighted imaging (PWI) represents salvageable brain tissue that will not evolve to complete cerebral infarction if blood flow is restored (2). The assumption is that penumbral tissue defined in this manner can be used to select patients for endovascular recanalization therapies that aim to restore blood flow (3). The mismatch hypothesis was recently tested in two large multicenter NIH-funded clinical trials, DEFUSE 2 (4) and MR RESCUE (5).

In both trials, tissue represented by MRI voxels was classified as at-risk or not at-risk based on diffusion and perfusion values. The DEFUSE 2 trial used the DPMM to define a “target profile” based on thresholds whereas the MR RESCUE trial used the DPMM to identify a “penumbral pattern” based on an equation. The two trials also differed in their conclusion with regard to the validity of the DPMM in identifying patient for endovascular therapy. DEFUSE 2 found that the DPMM could be used to identify patients who would have a good outcome with endovascular therapy. The DEFUSE 2 trial, however, did not investigate what would happen if patients with favorable MRI profiles were treated with medical therapy alone. MR RESCUE did not find that the DPMM could identify patients that would benefit from endovascular therapy. MR RESCUE did have a control group and thus was able to determine what would happen when patients who were thought to have at-risk tissue on MRI received medical therapy alone. One of the conclusions of the MR RESCUE trial was that a penumbral pattern on MRI conferred a better outcome but that endovascular therapy, even in the setting of a penumbral pattern, was of no additional benefit.

There are several potential reasons why these two trials led to opposite conclusions. Enrollment biases and differences in recanalization rates likely contributed. (6) However, the ability of these trials to identify a potential benefit of endovascular therapy, hinges on an accurate and unbiased estimate of at-risk tissue and core infarction. If the MRI measures used were inaccurate, then the entire premise upon which they are based falls apart. The dissimilar results of these two trials could be accounted for if the measures used were correct in one case and incorrect in the other. The negative results of the MR RESCUE could be explained if the definition overestimated at-risk tissue and under-estimated core infarction. The purpose of this study was to look at how the definition of at-risk tissue and core infarction differed between these two trials such that their results can be appropriately interpreted.

## Materials and Methods

Twelve patients who presented to our institution with acute ischemic stroke were retrospectively identified under an IRB approved protocol as having had an acute MRI scan with DWI and PWI for possible endovascular therapy. DWI and PWI were co-registered with an AIR linear transform using Diffeomap software (mristudio.org). Apparent diffusion coefficient (ADC) maps were calculated from B0 and B1000 source images using Matlab software (mathworks.com). Time-to-maximum (*T*_max_) maps were generated from the PWI source images after deconvolution of the arterial input function using a circular single value decomposition in Olea Sphere software (olea-medical.com). All subsequent processing to calculate the target profile and the penumbral pattern were done in Matlab.

For each MRI scan, a region of interest (ROI) was defined in the affected MCA territory as having a *T*_max_ value ≥2 s or an ADC value ≤700 μm^2^/s. ROIs were manually reviewed and edited to remove areas, which were artifactual such as the ventricular system. Then every voxel in the ROI was classified as being at-risk, core infarct, or neither for each of the definitions in the two studies. For the target profile (DEFUSE 2), at-risk was defined by voxels with ADC ≥ 600 μm^2^/s and *T*_max_ > 6 s and core infarct was defined as ADC < 600 μm^2^/s. Classification was based on absolute volumes and was not influenced by registration. For the penumbral pattern (MR RESCUE), at-risk tissue was defined by having a *T*_max_ > 2 s and by not being classified as core infarct. Core infarct in the penumbral pattern was defined by the equation 0.0044*ADC - 0.125*T_max_ – 0.902 ≥ 0. This definition of the penumbral pattern was used in the MR RESCUE trial until 2010 according to the published protocol (enrollment ran from 2004 to 2011).

Using these definitions, the volume of at-risk tissue and the volume of core infarct were calculated for each MRI. Using these volumes, a mismatch ratio, defined as the volume of at-risk tissue divided by the volume of core infarct, was generated for each definition. A percent core, defined as the proportion of the at-risk tissue which is core infarct, was generated for each. Additionally, the volume of tissue characterized by very low or absent blood flow (no-flow) was calculated for use in the classification of the target profile. This no-flow lesion was defined as tissue with *T*_max_ > 10 s.

An MRI was classified as having a penumbral pattern if it demonstrated a core infarct of ≤90 mL and a percent core of ≤70%. An MRI was classified as having a target profile if it demonstrated a core infarct <70 mL, a no-flow lesion <100 mL, and a mismatch ratio of ≥1.8.

The volumes of at-risk and core infarct tissue were compared between the two definitions using a paired *t*-test. Statistical analysis was done with the Stata software package (stata.com).

## Results

Of the 12 patients included in the analysis, 4 where female and their mean age was 68. Four of the patients had a known time of onset and had a mean time to MRI of 163 min. The remaining eight patients were wake-up strokes and had a mean time from wake-up to MRI of 193 min. All patients had a right middle cerebral artery (MCA) occlusion except for two, who had a left MCA occlusion, and one, who had a left posterior cerebral artery occlusion. Table 1 shows the salvageable tissue classifications of the 12 MRI scans, as well as at-risk volumes, core infarct volumes, percent cores, and mismatch ratios. Nine out of 12 patients (75%) where classified as having a penumbral pattern (MR RESCUE), while only 4 out of 12 patients (33%) were classified as having a target mismatch (DEFUSE 2). Of the nine patients classified as having salvageable tissue by the MR RESCUE trial, 5 (55%) would have been classified differently by the DEFUSE 2 trial.

**Table 1:** **At-risk tissue volumes, core infarct volumes, salvage classifications, percent cores, and mismatch ratios for each patient MRI scan in the study are displayed**.

MRI scan	At-risk volume (mL)		Core infarct volume (mL)		Classification		Percent core (%)		Mismatch ratio	
	Penumbral pattern	Target profile	Penumbral pattern	Target profile	Penumbral pattern?	Target profile?	Penumbral pattern	Target profile	Penumbral pattern	Target profile
1	75	58	5	6	Yes	Yes	7	10	13.8	9.7
2	7	3	1	3	Yes	No	20	120	5.0	0.8
3	25	12	9	17	Yes	No	37	150	2.7	0.7
4	121	40	7	24	Yes	No	6	59	16.5	1.7
5	58	23	8	14	Yes	No	14	62	7.2	1.6
6	46	33	48	84	No	No	103	255	1.0	0.4
7	61	62	47	41	No	No	77	66	1.3	1.5
8	33	10	2	3	Yes	Yes	7	27	14.9	3.7
9	160	93	35	36	Yes	Yes	22	38	4.6	2.6
10	47	31	7	28	Yes	No	15	90	6.7	1.1
11	25	21	19	20	No	No	75	92	1.3	1.1
12	103	47	13	14	Yes	Yes	13	31	7.8	3.3

**Figure 1:**
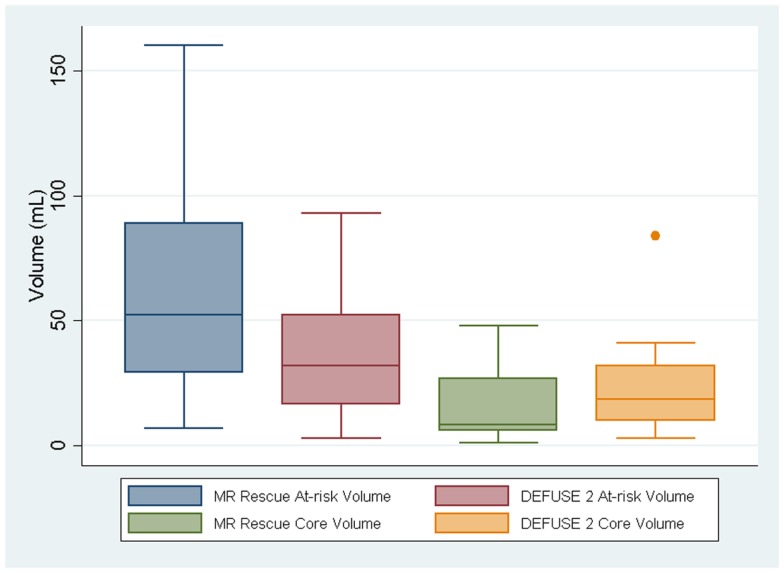
**The volumes of at-risk tissue and core infarct are displayed as a box plot for each trial**.

Although the at-risk volumes of the two definitions were correlated (*p* = 0.017), the penumbral pattern volume (mean ± SD = 63 ± 45 mL) was almost always larger than the target profile volume (mean ± SD = 36 ± 26 mL). The core infarct volumes on the other hand did not reach significance (*p* = 0.059). The target profile core infarct volumes (mean ± SD = 24 ± 22 mL) were almost always larger than the penumbral pattern core infarct volumes (mean ± SD = 17 ± 17 mL). Because of the way that the MR RESCUE defines at-risk tissue and core infarct, an increase in one is linked to a decrease in the other. There is no such a dependency between the two measures for the DEFUSE 2 definition. Figure 1 demonstrates that the MR RESCUE had larger volumes of at-risk tissue and smaller volumes of core infarct, while the DEFUSE 2 identified volumes that fell in between.

**Figure 2:**
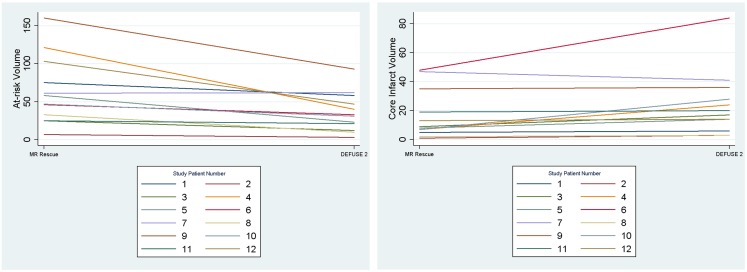
**The volumes of at-risk tissue and core infarct are plotted for each patient with lines connecting the values calculated by the two methods (MR RESCUE and DEFUSE 2)**. This demonstrates that the rank order of volumes assigned by the definitions is different.

**Figure 3:**
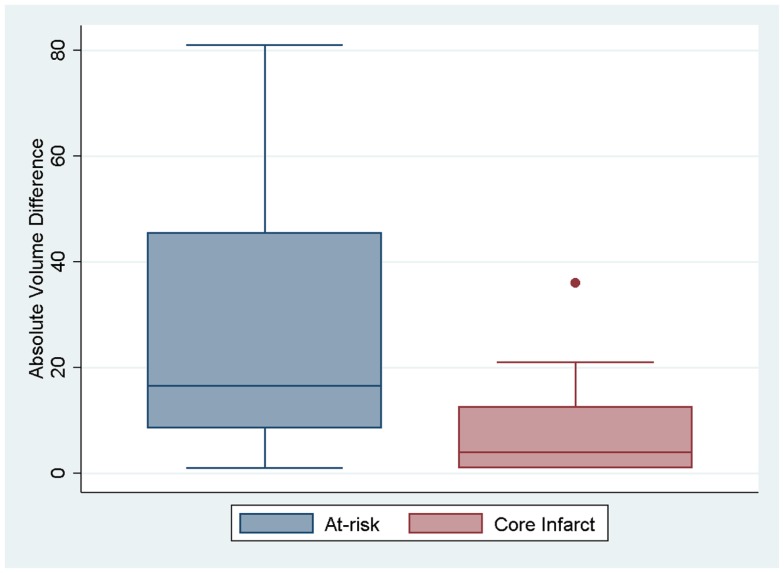
**Box plots showing the absolute difference between the two definitions for at-risk tissue and core infarct are displayed**.

**Figure 4:**
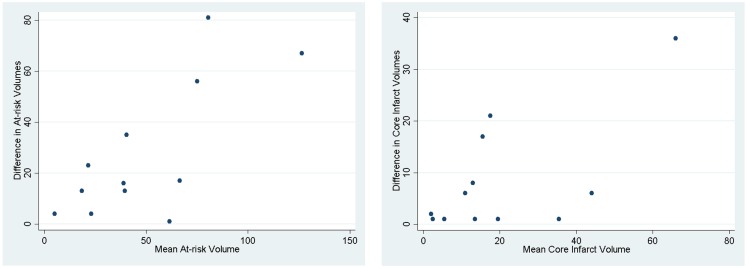
**Bland–Altman plots demonstrating how the two methods designed to measure the same parameter have different results**. The mean of the two methods is plotted against the difference in the two methods.

**Figure 5:**
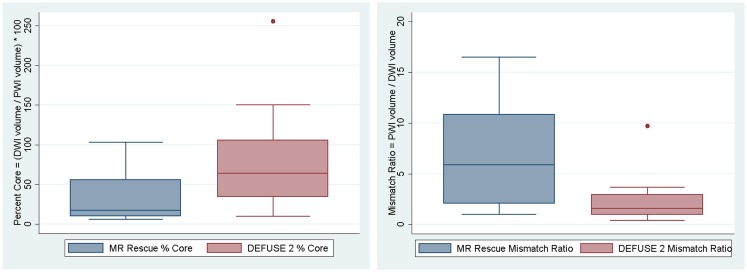
**The percent core and mismatch ratios for each trial are displayed as a box plot**.

We assessed if the differences in both at-risk and core infarct volume between the two trials are a result of a bias being introduced by one of the definitions, which would be captured by an offset. The at-risk volumes and core infarct volumes for each patient as defined by the two trials are plotted next to each other and connected by a line in Figure 2. Figure 2 demonstrates that the rank order is different between the two definitions as the lines frequently cross. Figure 3 is a box plot of the absolute difference in volumes between the definitions demonstrating that the differences between the two definitions are themselves variable. Figure 4 shows Bland–Altman plots comparing the mean volume measured by the two methods with the difference in volume measured by the two methods. This type of analysis is used for judging two methods designed to measure the same parameter. In this case, it demonstrates that for at-risk tissue, and to a lesser extent core infarct, the two methods rarely agree and the difference is more pronounced at higher volume measurements. For at-risk volumes, the scatter points fall mostly above the diagonal while for core infarct volumes the scatter points fall mostly below the diagonal, which demonstrates that the two definitions affect the at-risk and core infarct volumes in opposite directions. This difference in the salvageable tissue estimation for the two trials is further amplified when percent core and mismatch ratios are calculated. The mean percent core, which can be thought of as the percent of the perfusion deficit that has infarcted, for the penumbral pattern, was 33 ± 33%, while for the target profile it was 83 ± 67%. The mean mismatch ratio for the target profile was 2.3 ± 2.5, while for the penumbral pattern it was 6.9 ± 5.5% (Figure 5).

## Discussion

The role of endovascular therapy in the management of acute stroke remains controversial. Anecdotal experience tells us that endovascular therapy can be effective in some cases. Three recent randomized clinical trials of endovascular recanalization therapy have failed to demonstrate a benefit (5, 7, 8) Without a positive clinical trial, it seems unlikely that endovascular therapy will remain a treatment option. One of these trials, MR RESCUE, may appear to some as an example of why MRI-based patient selection should not be part of future clinical trials. However, if the MR RESCUE definition of penumbra is flawed, then we may wrongly discard a tool that could help identify a subset of patients for whom endovascular therapy may indeed be effective. Thus it is very important that the calculations used to identify salvageable tissue are carefully scrutinized.

In this study, the salvageable tissue profiles for the MR RESCUE and DEFUSE 2 trials were compared and contrasted in 12 new patients evaluated at our center. The penumbral pattern (MR RESCUE) consistently identified larger volumes of tissue at risk (therefore possibly over-estimating the potential benefit from recanalization) compared to the target profile (DEFUSE 2). Additionally, the penumbral pattern consistently identified a smaller core ischemic volume than the target profile. The importance of the volume of the ischemic core is gaining appreciation in the literature. (9–11) Unlike the core defined by the penumbral pattern, the core defined by the target profile has been tested in other populations. (12) Not only does the penumbral pattern consistently identify a smaller ischemic core, but the volume threshold for the core, above which a patient is unlikely to respond to therapy, was higher for MR RESCUE (90 vs 70 mL for DEFUSE 2). The differences in the definition of the ischemic core could also have played a part in different outcomes between the trials.

MR RESCUE, therefore, had a higher probability of defining any give patient’s tissue as salvageable compared to DEFUSE 2. Specifically, the penumbral pattern of the MR RESCUE trial likely included larger core volumes, no-flow lesions, smaller mismatches, and smaller penumbras. This could explain the lack of association between endovascular therapy and good outcome in this trial.

There are several limitations to this study. This was a small sample of patients which may not be representative of the patients enrolled in the trials discussed. Specifically, patients with unknown time of onset were not included in either trial. This analysis demonstrates how one small population would be differently classified by the two methods, but is not powered such that it can be generalized to the specific patients enrolled in the two trials. Additionally, the two MRI analysis methods were replicated manually and may not fully represent the automated way MRIs were processed in each trial.

Although the analysis presented here does not tell us how specific patients enrolled in the two trials would have been differently classified by the two methods, it does indicate that the results of the MR RESCUE trial should not be considered conclusive evidence against a role of MRI in the selection of patients for endovascular therapy. The DEFUSE 2 trial will need to be repeated but with the addition of a control group who have a salvageable MRI pattern but do not receive endovascular therapy. MR RESCUE trial provides the equipoise needed to conduct such a trial.

## Conflict of Interest Statement

The authors declare that the research was conducted in the absence of any commercial or financial relationships that could be construed as a potential conflict of interest.
